# Effects of phone versus mail survey methods on the measurement of health-related quality of life and emotional and behavioural problems in adolescents

**DOI:** 10.1186/1471-2458-9-491

**Published:** 2009-12-30

**Authors:** Michael Erhart, Ralf M Wetzel, André Krügel, Ulrike Ravens-Sieberer

**Affiliations:** 1 Department of Psychosomatics in Children, Center for Obstetrics and Pediatrics, University Medical Center Hamburg-Eppendorf, Building W 29, Martinistr 52, D-20246 Hamburg, Germany

## Abstract

**Background:**

Telephone interviews have become established as an alternative to traditional mail surveys for collecting epidemiological data in public health research. However, the use of telephone and mail surveys raises the question of to what extent the results of different data collection methods deviate from one another. We therefore set out to study possible differences in using telephone and mail survey methods to measure health-related quality of life and emotional and behavioural problems in children and adolescents.

**Methods:**

A total of 1700 German children aged 8-18 years and their parents were interviewed randomly either by telephone or by mail. Health-related Quality of Life (HRQoL) and mental health problems (MHP) were assessed using the KINDL-R Quality of Life instrument and the Strengths and Difficulties Questionnaire (SDQ) children's self-report and parent proxy report versions. Mean Differences ("d" effect size) and differences in Cronbach alpha were examined across modes of administration. Pearson correlation between children's and parents' scores was calculated within a multi-trait-multi-method (MTMM) analysis and compared across survey modes using Fisher-Z transformation.

**Results:**

Telephone and mail survey methods resulted in similar completion rates and similar socio-demographic and socio-economic makeups of the samples. Telephone methods resulted in more positive self- and parent proxy reports of children's HRQoL (SMD ≤ 0.27) and MHP (SMD ≤ 0.32) on many scales. For the phone administered KINDL, lower Cronbach alpha values (self/proxy Total: 0.79/0.84) were observed (mail survey self/proxy Total: 0.84/0.87). KINDL MTMM results were weaker for the phone surveys: mono-trait-multi-method mean r = 0.31 (mail: r = 0.45); multi-trait-mono-method mean (self/parents) r = 0.29/0.36 (mail: r = 0.34/0.40); multi-trait-multi-method mean r = 0.14 (mail: r = 0.21). Weaker MTMM results were also observed for the phone administered SDQ: mono-trait-multi-method mean r = 0.32 (mail: r = 0.40); multi-trait-mono-method mean (self/parents) r = 0.24/0.30 (mail: r = 0.20/0.32); multi-trait-multi-method mean r = 0.14 (mail = 0.14). The SDQ classification into borderline and abnormal for some scales was affected by the method (OR = 0.36-1.55).

**Conclusions:**

The observed differences between phone and mail surveys are small but should be regarded as relevant in certain settings. Therefore, while both methods are valid, some changes are necessary. The weaker reliability and MTMM validity associated with phone methods necessitates improved phone adaptations of paper and pencil questionnaires. The effects of phone versus mail survey modes are partly different across constructs/measures.

## Background

Telephone interviews have become established as an alternative to traditional mail surveys for collecting epidemiological data in public health research. In comparison to mail surveys, telephone interviews often allow more inexpensive data collection [1-3], improve the completeness of the data records collected [4-6] and are usually characterised by higher response rates [7-9]. In addition, they allow an inventory of questionnaires to be adapted more flexibly to different specific circumstances or key issues of interest and allow the data to be analysed very promptly since they are available immediately after completing the interview. The two methods of carrying out interviews are often combined within a single study so as to optimally adapt the data collection process to the underlying conditions associated with the specific study [10-12]. However, the use of different data collection methods raises a question: to what extent do the results of telephone and mail surveys correlate with each other? Differences in results between telephone and mail surveys have already been demonstrated in numerous areas of interest and in the instruments used to collect data about them, such as surveys of patient satisfaction [13,14], alcohol and drug abuse [15,16] or mental health [17,18]. Different response rates between postal and phone surveys could result in biased samples that impact the data quality, and the different survey modes may also affect the response behaviour itself. This impact, however, is dependent on the actual content of the questions. Previous studies have found that face-to-face or telephone surveys may result in a tendency towards less reported morbidity, health care utilisation or socially inadequate behaviour [4,16,19]. These results were mainly attributed to the reduced anonymity of face-to-face or phone surveys. Similarly, other studies have found more positive assessments of mental health dimensions of health-related quality of life when gathered by telephone rather than by mail survey [2,20,21].

With regards to children and adolescents, there is a lack of empirical studies about differences between postal and phone survey methods for determining their self-reported health and also for the still widely-used parent proxy reports. With the increasing trend of public health research on children and adolescents, such knowledge is important. In Germany, the representative adolescent health surveys that have been carried out by mail since the mid 1980s by the Robert Koch Institute, as well as the German Health Interview and Examination Survey of Children and Adolescents (KiGGS), its Mental Health Module (BELLA study), and their follow-ups have now been augmented by additional representative telephone surveys [22-24]. Hence, it is important to assess the influence of the survey method used on the data collected. We do not know how the assessment of different psychosocial constructs is influenced by survey modes, but this issue is important to consider to correct for such deviations if necessary.

One of the outcomes determined by these federal health surveys is the concept of health-related quality of life (HRQoL), which takes into account physiological, emotional, mental and social dimensions in subjectively perceived aspects of health [25] and which has firmly established itself as an integral component of health research. HRQoL denotes, in psychological terminology, a multidimensional construct covering physical, emotional, mental, social and behavioural components of well-being and function as perceived by patients and/or other observers [26]. To assess adolescents' health-related quality of life over the telephone, the KINDL-R Questionnaire for Measuring Health-related Quality of Life in Children and Adolescents [27,28] is used.

Emotional and behavioural problems constitute another important outcome surveyed in the federal health surveys. The Strengths and Difficulties Questionnaire (SDQ) [29] is currently used to assess emotional (depressed mood, anxiety), conduct (aggressive and antisocial behaviour) and peer problems (social contact difficulties) as well as hyperactivity (hyperactivity, inattention) in respondents over the phone. The constructs assessed by the SDQ are based on the ICD-10 classification of diseases. However, the SDQ does not allow the establishment of ICD 10 diagnoses. Positive attitudes and prosocial behaviour are also assessed by this method.

Both the KINDL and the SDQ are widely used internationally and are well validated [30,31]. However, to the best of our knowledge, studies comparing the administration of an interview by telephone or by mail have so far not been carried out for either of these instruments. The present study thus set out to study possible effects of telephone and mail survey methods on the KINDL-R and the SDQ in a random German sample of adolescents and their parents.

This paper aims to add to the current knowledge on differences attributable to different survey modes, their potential impact on test results, and ways to deal with these concerns. In addition to looking at the composition of the sample, this comparison focuses especially on the psychometric properties of the test data with regards to any differences in means and variances and internal consistency, as well as convergent and discriminant validity resulting from the different survey methods. It especially compares the influence of survey methods on the assessment of two different constructs: health-related quality of life versus emotional and behavioural problems.

## Methods

### Sample

The data on which this study is based were collected in the context of a nation-wide survey on the equivalence of aspects of health between children and their parents, carried out by telephone and by mail. In collaboration with the Centre for Survey Research and Methodology (ZUMA), a two-step, stratified, list-based, random sample of adults living in Germany with children aged 8 to 17 was drawn. Four thousand families were selected at random from all the households officially registered in 42 German municipalities, preselected on the basis of region, population figures and a cost-of-living classification. Inclusion criteria were sufficient understanding of the German language.

### Procedure

The survey was carried out as a randomised cross-sectional study. The selected households were randomly assigned to being surveyed by mail (2000 households) or by telephone (2000 households). Data collection lasted from May 2003 until March 2004. The survey was carried out in line with the recommendations issued by McColl et al. [8]. All procedures were carried out following the data protection requirements of the European Parliament (Directive 95/46/EC of the European Parliament and of the Council of 24 October 1995 on the protection of individuals with regard to the processing of personal data and on the free movement of such data). The study was approved by the ethics committee of the Federal Robert Koch Institution where the work was carried out. Informed consent was collected from the parents and the children.

All households assigned to the **mail survey sample **received a personalised letter informing them of the institution carrying out the interview and about the interview's contents and were asked to complete the enclosed questionnaires for one parent and the selected child. In case of households with more than one eligible child, the nearest birthday criterion guided the selection of the child. Each household was provided with a stamped envelope for returning the questionnaire. After two and four weeks, identical reminders were sent to households that had not replied.

The telephone numbers of the households in the **telephone survey sample **were researched from telephone directories using appropriate address data. Different methods of contacting households were chosen depending on whether or not the household was listed in the directory. All 2000 households received a personalised letter in advance, informing them of the institution carrying out the interview and about the interview's contents. Households whose numbers were recorded in a public directory (53.6%) were told that the study centre would be phoning them within the next 14 days. No telephone number could be determined for 928 households (46.4%). These households were asked to supply their telephone number to the study centre. A stamped return envelope was included with the letter for this purpose. Households that failed to respond were reminded twice in writing at 2-week intervals. One hundred sixty-eight unlisted households supplied their telephone numbers in this way. Overall, telephone numbers were available for 1240 households (62.0%).

Computer-assisted telephone interviews (CATI) were conducted mainly between 3 and 8 p.m. to increase the chances of reaching working adults. Specially trained interviewers contacted the households 1-2 weeks after the letter of information was sent out or after receiving the telephone number. Up to 12 attempts were made to contact the respondents. The parents and children in a family were interviewed separately from one another. The interviewers read aloud the time frame of the item, the item statement and the item answer categories for every item.

### Measures

The children's and adolescents' health-related quality of life was assessed using the KINDL-R questionnaire on health-related quality of life [10], which has been previously tested in epidemiologic studies with regards to its psychometric properties as a quality of life screening instrument [13]. The KINDL-R questionnaire is a generic and revised instrument in the German language for the assessment of health-related quality of life that can be used in clinical as well as in healthy populations of children and adolescents. The KINDL-R questionnaire consists of 24 items covering six dimensions of quality of life (referring to the past week): Physical well-being (e.g., ...I have felt sick), Emotional well-being (e.g., ...I have felt fearful or insecure), Self-worth (e.g., ....I was happy with myself), Well-being in the family (e.g., ...I felt comfortable at home), Well-being related to friends/peers (e.g....I got along with my friends), and School-related well-being (e.g....I was afraid of getting bad grades). Each item provides five answer categories: never, seldom, sometimes, often and always. Item responses were coded with values between 1 and 5, with higher values indicating "better" quality of life ratings. The four item scores per dimension were added and transformed into values between 0 and 100 points, with a larger score indicating a better quality of life. Furthermore, a total score for health-related quality of life was calculated from all 24 items, which also ranges between 0 and 100 points. The KINDL-R questionnaire includes both a child and adolescent self-assessment version and an external assessment version to be completed by the parents.

The SDQ [29], a brief behavioural screening questionnaire for children and teenagers, surveys mental health symptoms and positive attitudes. The current study applied the adolescents' self-report version for the 13-18 year olds and the parents' proxy report version for all participants. Both versions assess positive or negative attributes with 25 items focusing on the following dimensions: Emotional symptoms (e.g., I am often unhappy, down-hearted or tearful), Conduct problems (e.g., I get very angry and often lose my temper), Hyperactivity/inattention (e.g., I am constantly fidgeting or squirming), Peer relationship problems (e.g., I get on better with adults than with people my own age) and Prosocial behaviour (e.g., I am helpful if someone is hurt, upset or feeling ill). Each item is scored on a 3-point scale with 0 = not true, 1 = somewhat true, and 2 = certainly true, with higher scores indicating larger problems, except for with regards to Prosocial behaviour questions, in which a higher score indicates more positive behaviour. The item scores were summed to obtain subscores ranging from 0-10. Items related to the four problem areas were then summed to generate a Total difficulty score (0-40). The SDQ has been translated and validated in several countries. From a large representative sample of the United Kingdom, cut-off points have been defined classifying the test results into normal, borderline and abnormal mental health problem scores [32]. The German translation of the SDQ has been applied, tested and validated in several studies [33,34].

In addition to the KINDL-R and the SDQ, the parents' questionnaire also included questions about their own health behaviour, physical and emotional complaints and self-assessed health-related quality of life. Furthermore, socio-demographic data (age, sex, marital status, work status) and details of socio-economic status (education) and acceptance were also collected. Similarly, the children's questionnaire included additional questions about their own health behaviour, as well as physical and emotional complaints. Socio-demographic data (age, sex), details of socio-economic status (Family Affluence Scale - FAS [35]) and the acceptance of the particular survey method were also collected.

### Statistical Analyses

The socio-demographic and socio-economic characteristics of subjects interviewed over the telephone were compared with those of subjects interviewed by mail, and χ^2 ^tests were used to test for significant differences. The effects of the method of administration were examined by comparing the means of the KINDL and SDQ scales using an analysis of variance. Whether the different survey methods yielded different effects for gender, age group (8-12 vs. 13-18), or different sources of information was determined: Statistical interaction terms were specified and included as additional sources of variation in multifactor ANOVAs. Differences in the distributions of the responses were investigated using Levene tests.

The internal consistency of item responses (Cronbach alpha) was calculated separately for the two methods of data collection. A multi-trait-multi-method analysis was performed for the two survey methods to test convergent and discriminant validity [36]. Pearson correlations were calculated between corresponding self and proxy scores (mono-trait-multi-method block), between the self scores and between the proxy scores (multi-trait-mono-method block), and between non-corresponding self and proxy scores (multi-trait-multi-method block). Fisher-Z transformations were applied to the calculated mean correlations per block to judge the magnitude of difference in correlation (Fisher-Z 0.10-0.29 = "small"; 0.3-0.49 = "medium"; 0.5 and higher = "large").

To test whether the survey modes led to different results regarding the classification of the SDQ, the frequencies of the normal-borderline-abnormal classification were cross-tabulated against the survey mode. A multinomial logistic regression analysis was performed to test for statistically significant differences.

Although some of the analyses involved multiple tests of the same hypothesis, the alpha level was not adjusted since this would have increased the risk of failing to identify a population-wide difference. An alpha level of 0.05 was used. Based on previous studies with the HRQoL instrument SF-36 in adolescents, a difference of 0.2 standard deviations, equivalent to a "small" Cohen's "d" effect size of 0.2 [37], was expected. Enabling a statistical power of 0.80 to detect such an effect with an alpha level of p = 0.05 requires n = 394 respondents to be surveyed under each mode of administration. The actual sample size of more than 1700 data sets thus permits analyses on the entire sample, as well as samples stratified for sex or for two equal-sized age groups. The effect size measure Cohen's "d" [37] was calculated to enable comparison of effects between different strata and the entire sample independent from the actual statistical power of the analyses, which depends on the sample-size. For analyses stratified for more than two strata, the interpretation focused on these effect sizes rather than on mere statistical significance. Effect sizes of "d" = 0.20-0.49 were classified as "small"; 0.50-0.79 as "medium" and 0.80 and greater as "large".

As cases with missing values were excluded for each separate analysis (pairwise), the numbers reported in the results differ from those reported in the sample description.

## Results

### Sample and Response

Mail delivery of the survey documents was possible in 1928 of 2000 cases (96.4%). Establishing a valid telephone number and making telephone contact was only possible for 1066 households in the telephone sample (53.3%). The completion rate of the mail sample was 45.8% (916 cases) and that of the telephone sample was 41.3% (825 cases). The deficits in the ability to contact households by telephone were almost completely balanced by the distinctly higher willingness of respondents contacted by telephone to participate in the survey. Taking into account the n = 72 households with invalid address (neutral drop-out in mail survey) and the n = 70 households were it was not possible to get through to someone by phone within the scheduled survey time (neutral drop out in phone survey), the response rate was 47.5% in the mail survey, compared with 42.7% in the telephone survey.

Table 1 shows that the populations of the two samples did not differ in terms of age and gender distributions. The two parent groups of respondents were on average 41.7 years old with a standard deviation of about 5.5 years. More than 80%, were mothers, both in the telephone and in the mail interviews. No statistically significant differences were apparent in terms of parent respondents' marital status, education or work status. The mean age of the children was 13.4 and 13.3 years, for mail and telephone, and was not statistically significant different between the groups. The small difference in the proportion of girls between samples was not statistically significant. Thus, the samples were comparable with regards to the basic socio-demographic and socio-economic variables.

**Table 1:** Socio-demographic characteristics of the sample depending on administration mode

	**Mail survey**		**Phone survey**	
		
Parental Mean age (sd)	41.70 (5.48)		41.74 (5.49)	
t - value	-.166 p = .868			
Parental status	n	%	N	%
Mother	755	83.6	655	82.8
Father	137	15.2	130	16.4
Other	11	1.2	6	0.8
χ^2 ^- value _(*df *= 2)_	0.727 p = .695			
Marital status	n	%	N	%
married	688	76.4	644	81.4
widowed	9	1.0	7	0.9
living apart	97	10.8	78	9.9
divorced	35	3.9	23	2.9
single	71	7.9	39	6.5
χ^2 ^- value _(*df *= 4)_	8.568 p = .073			
Status of education	n	%	N	%
no graduation	20	2.2	8	1
elementary school	211	23.4	165	20.9
secondary school level	372	41.2	333	42.2
University entrance diploma	116	12.8	111	14.1
grad. of advanced technical college	82	9.1	70	8.9
university degree	102	11.3	103	13
χ^2 ^- value _(*df *= 5)_	6.477 p = .263			
Occupational Status	n	%	N	%
full time	319	35.5	267	33.8
part time	350	39.0	311	39.3
casual labour	37	4.1	47	5.9
out of work	35	3.9	32	4.0
homemaker/house husband	138	15.4	113	14.3
student/retiree	10	1.1	13	1.6
miscellaneous	9	1.0	8	1.0
χ^2 ^- value _(*df *= 6)_	4.420 p = .620			
Childrens mean age (sd)	13.38 (2.79)		13.32 (2.88)	
t - value	.486 p = .627			
Gender of the Child	n	%	n	%
Female	464	50.9	401	48.6
Male	448	49.1	424	51.4
χ^2 ^- value _(*df *= 1)_	.894 p = .344			

Cases with sociodemographic and socio-economic information available

### Psychometric Differences for the KINDL-R

Table 2 shows that phone survey respondents on average scored higher on the KINDL self-report. The observed differences were statistically significant for the Total and the subscales Psychological, Friends, and School. The magnitude of the difference was small, as the "d" effect size ranged from 0.04 to 0.20. These effects were slightly more pronounced for 13-18 years olds ("d" = 0.07-0.24). For the subscale Self-Esteem, the statistical interaction between age and survey method was statistically significant.

**Table 2:** KINDL-R Test score distribution across modes of administration

	Mail		phone		Δ-methods				
					All	Girls	Boys	8-12	13-18
	**Mean**	**SD**	**Mean**	**SD**	**effect size "d" ^stat. sign Δ-Mean; stat. sign Δ-SD^**				

*Children's self-report*	*n = 891-895*^*a*^		*n = 769-777*^*a*^		*N = 1660-1672*^*a*^	*N = 832-837*^*a*^	*N = 824-832*^*a*^	*N = 645-649*^*a*^	*N = 1015-1026*^*a*^
Physical	75.70	16.20	75.87	15.99	0.01^ns,ns^	-0.08^ns,ns^	0.11^ns,ns^	-0.02^ns,ns^	0.07^ns,ns^
Psychological	80.74	13.87	82.83	12.90	0.16**^,ns^	0.15*^,ns^	0.15*^,ns^	0.13^ns,ns^	0.19**^,^
Self esteem	61.26	18.95	62.02	17.04	0.04^ns,^**	-0.02^ns,ns^	0.10^ns,ns^	-0.12^ns,ns^	0.15*^,^**
Family	82.82	14.92	83.79	16.13	0.06^ns,ns^	-0.09^ns,^**	0.22**^;ns^	0.05^ns,ns^	0.08^ns,^*
Friends	76.50	16.34	79.50	13.71	0.20**^,^**	0.22**^,^**	0.16*^,^*	0.15^ns,ns^	0.25**^,^**
School	68.31	17.99	71.36	17.67	0.17**^,ns^	0.12^ns,ns^	0.22**^,ns^	0.23**^,ns^	0.21**^,ns^
Total Qol	74.23	11.01	75.88	9.90	0.16*^,^*	0.06^ns,ns^	0.25**^,ns^	0.09^ns,ns^	0.24**^,ns^
*Parent's proxy-report*	*n = 895-905*^*a*^		*n = 759-791*^*a*^		*N = 1654-1696*^*a*^	*N = 822-842*^*a*^	*N = 828-85*^*a*^	*N = 661-66*^*a*^	*N = 992-103*^*a*^
Physical	81.79	15.61	85.19	15.38	0.22**^,ns^	0.17*^,ns^	0.27**^,ns^	0.17*^,ns^	0.26**^;ns^
Psychological	80.26	13.60	83.75	12.29	0.27**^,^**	0.23**^,ns^	0.30**,**	0.17*^,ns^	0.33**^;^**
Self esteem	66.62	15.05	71.49	14.83	0.19**^,ns^	0.21**^,ns^	0.17*^,ns^	0.14^ns,ns^	0.24**^;ns^
Family	77.61	14.28	78.31	14.19	0.05^ns,ns^	0.03^ns,ns^	0.07^ns,ns^	-0.02^ns,ns^	0.09^ns,ns^
Friends	77.16	14.62	77.66	14.07	0.03^ns,ns^	0.08^ns,ns^	-0.02^ns,ns^	-0.05^ns,ns^	0.09^ns,ns^
School	73.41	16.22	76.18	15.64	0.17**^,ns^	0.17*^,ns^	0.18**^,ns^	0.11^ns,ns^	0.24**^,ns^
Total Qol	76.46	10.47	78.81	9.69	0.23**^,^*	0.22**^,ns^	0.24**^,ns^	0.13^ns,ns^	0.31**^,ns^

^a ^Numbers vary due to missing values in the dimensions.

^b ^effect size classification: 0.20 = small; 0.50 = medium; 0.80 = large

^ns ^non significant; * p < .05; ** p < .01; first entry = t-test for mean differences; second entry = Levene test for SD-differences (all statistically significant SD-differences had the same direction as indicated by data-column 2 and 4)

Higher KINDL scores for the phone survey methods were also observed for the parent proxy version. Statistically significant differences were observed for the proxy reported KINDL Total and the subscales Physical, Psychological, Self-esteem and School. The magnitude of the difference could be classified as small effects ("d" = 0.03-0.27). These differences were slightly more pronounced than for the self-report version. A statistically significant interaction between survey method and the source of information (self vs. proxy) was observed for the scales Physical and Peers.

There was a tendency towards a smaller dispersion of test scores in the phone survey compared to the mail survey. For the self-report scales Self-esteem, Friends and the Total, as well as the proxy report scales Psychological and the Total, the SD-difference reached statistical significance.

With respect to internal consistency in the mail survey, Table 3 shows that Cronbach alpha was 0.84(0.87) for the KINDL Total self(proxy) report scale and ranged from 0.60(0.64) to 0.72(0.75) for the self(proxy) report subscales. For the phone survey, lower Cronbach alphas of 0.79(0.84) were observed for the self (proxy) report KINDL Total and the subscales (self-report = 0.40-0.62; proxy report = 0.51-0.68).

**Table 3:** Cronbach alpha and correlation between KINDL-R test scores (child's self-report and parent proxy report)

		*Children's self-report*						*Parent's proxy-report*					
		PHY	PSY	SEL	FAM	FRI	SCH	PHY	PSY	SEL	FAM	FRI	SCH
		C1	C2	C3	C4	C5	C6	P1	P2	P3	P4	P5	P6
	*alpha*	*0.58*	*0.51*	*0.62*	*0.74*	*0.40*	*0.52*	*0.68*	*0.57*	*0.68*	*0.66*	*0.57*	*0.51*

C1	*0.61*		0.38	0.28	0.30	0.25	0.32	**0.37**	0.23	0.22	0.15	0.11	0.21
C2	*0.62*	0.41		0.22	0.33	0.41	0.30	0.19	**0.26**	0.13	0.16	0.19	0.13
C3	*0.72*	0.36	0.38		0.23	0.31	0.20	0.09	0.16	**0.20**	0.11	0.07	0.13
C4	*0.71*	0.25	0.38	0.26		0.23	0.30	0.14	0.17	0.16	**0.32**	0.08	0.12
C5	*0.60*	0.32	0.48	0.35	0.25		0.22	0.13	0.20	0.15	0.10	**0.26**	0.06
C6	*0.60*	0.44	0.36	0.33	0.32	0.24		0.13	0.16	0.18	0.16	0.01	**0.41**

P1	*0.67*	**0.46**	0.23	0.22	0.12	0.19	0.18		0.48	0.39	0.23	0.28	0.28
P2	*0.69*	0.30	**0.35**	0.25	0.27	0.34	0.21	0.47		0.53	0.39	0.36	0.40
P3	*0.71*	0.30	0.27	**0.34**	0.23	0.26	0.28	0.39	0.56		0.37	0.42	0.37
P4	*0.75*	0.16	0.18	0.17	**0.46**	0.14	0.18	0.29	0.48	0.43		0.22	0.33
P5	*0.70*	0.22	0.28	0.22	0.18	**0.51**	0.07	0.32	0.53	0.42	0.34		0.18
P6	*0.64*	0.29	0.26	0.20	0.20	0.15	**0.54**	0.30	0.39	0.41	0.31	0.24	

Lower triangular matrix = mail; upper triangular matrix = phone administration

^a ^statistically significant deviation after Fisher-Z Transformation

PHY = Physical; PSY = Psychological; SEL = Self-Esteem; FAM = Family; FRI = Friends; SCH = School;

Alpha for the self-report total was 0.84 mail vs. 0.79 phone;

Alpha for the parent proxy report total was 0.87 mail vs. 0.84 phone;

Correlation between self-report and proxy report total Score was r = 0.54 (mail) and 0.39 (phone)

For the KINDL, MTMM analysis revealed lower convergent and discriminant validity for the phone survey methods. The average mono-trait-multi-method correlation (between corresponding self-report and proxy report scores) was r = 0.45 (range = 0.34-0.54) for the mail survey and r = 0.31 (range = 0.20-0.41) for the phone survey. The actual differences in r ranged from 0.08 to 0.25. Expressed in Fisher-Z, these differences ranged from 0.09 to 0.29. The average multi-trait-mono-method correlation (across the self-report/parent report version) was r = 0.34/0.40 for the mail survey, while for the phone survey, it was r = 0.29/0.35 (self-report/parent report) on average and in many instances similar or even higher than the desirable mono-trait-multi-method correlations. The average multi-trait-multi-method correlation between non-corresponding self-and proxy- scales was r = 0.21 for the mail survey and r = 0.14 for the phone survey. (Table 3). The correlation between the self-report and proxy report Total Qol score was r = 0.54 for the mail and r = 0.39 for the phone survey.

### Psychometric Differences for the SDQ

Table 4 shows that on average the phone survey yielded lower problem ratings on the SDQ self-report version. The observed differences were statistically significant for the Total difficulties score and for the Emotional, Conduct and Peer problem subscales. More Prosocial behaviour was reported on the phone. The magnitude of these differences could be classified as small effects, with "d" effect sizes between 0.04 and 0.20 (Prosocial: -0.32). These effects were slightly more pronounced for girls ("d" = 0.10-0.23; Prosocial: -0.39) than for boys ("d" = 0.03-0.18; Prosocial: -0.28). A statistically significant interaction between survey method and gender was observed for Hyperactivity.

**Table 4:** SDQ Test score distribution across modes of administration

	Mail		Phone		Δ-methods				
					All	Girls	Boys	8-12	13-18
	**Mean**	**SD**	**Mean**	**SD**	**effect size "d" ^stat. sign Δ-Mean; stat. sign Δ-SD^**				

*Children's self-report*^*b*^	*n = 528-530*^*a*^		*n = 498*		*N = 1026-1028*^*a*^	*N = 520-522*^*a*^	*N = 504*		
Emotional	2.48	2.04	2.19	1.87	0.15*^,^*	0.15^ns,ns^	0.15^ns,ns^	^d^	^d^
Conduct	1.87	1.28	1.65	1.24	0.17**^,ns^	0.21*^,ns^	0.13^ns,ns^	^d^	^d^
Hyperactivity	3.48	2.15	3.39	2.16	0.04^ns,ns^	0.10^ns,ns^	-0.03^ns,ns^	^d^	^d^
Peer Problems	2.07	1.54	1.78	1.33	0.20**^,^*	0.21*^,^*	0.18*^,ns^	^d^	^d^
Prosocial^c^	7.70	1.59	8.22	1.61	-0.32**^,ns^	-0.39**^,ns^	-0.28**^,ns^	^d^	^d^
Total	9.89	4.55	9.01	4.50	0.19**^,ns^	0.23**^,ns^	0.14^ns,ns^	^d^	^d^
*Parent's proxy-report*^*b*^	*n = 888-900*^*a*^		*n = 791*		*N = 1679-1691*^*a*^	*N = 833-839*^*a*^	*N = 842-848*^*a*^	*N = 657-662*^*a*^	*N = 1022-1029*^*a*^
Emotional	1.57	1.70	1.64	1.85	-0.05^ns,ns^	-0.06^ns,ns^	-0.04^ns,ns^	-0.13^ns,ns^	0.01^ns,ns^
Conduct	1.69	1.51	1.88	1.50	0.13**^,ns^	0.11^ns,ns^	0.14*^,ns^	0.05^ns,ns^	0.20**^,ns^
Hyperactivity	2.76	2.26	2.77	2.31	-0.01^ns,ns^	-0.01^ns,ns^	0.01^ns,ns^	0.02^ns,ns^	-0.04^ns,ns^
Peer Problems	1.43	1.68	1.26	1.47	0.11*^,^**	0.13^ns,^**	0.09^ns,ns^	0.12^ns,ns^	0.11^ns,^**
Prosocial^c^	7.85	1.81	8.39	1.54	-0.31**^,^**	-0.34**^,^**	-0.31**^,^**	-0.26**^,^**	-0.35**^,^**
Total	7.44	5.15	7.55	5.14	-0.02^ns,ns^	-0.02^ns,ns^	-0.02^ns;ns^	-0.01^ns,ns^	-0.04^ns,ns^

^a ^Numbers vary due to missing values in the dimensions.

^b ^effect size classification: 0.20 = small; 0.50 = medium; 0.80 = large

^ns ^non significant; * p < .05; ** p < .01; first entry = t-test for mean differences; second entry = Levene test for SD-differences (all statistically significant SD-differences had the same direction as indicated by data-column 2 and 4)

^b ^Emotional = Emotional Symptoms; Conduct = Conduct Problems; Prosocial = Prosocial Behaviour; Total = Total Difficulties Score

^c ^Higher values indicate more strengths

^d ^SDQ self-report was administered for the 13-18 year olds only.

Differential effects were observed for parent proxy version of the phone survey. A statistically significant interaction between the survey method and the source of information (self vs. proxy) was observed for the scales Conduct and Total. While the self-report phone survey yielded lower problem ratings, parents reported more problems over the phone. Statistically significant differences between survey methods were observed for the Conduct subscale, with higher problem ratings via phone, while for Peer problems and Prosocial less problems and more Prosocial behaviour were reported via phone. The magnitude of the difference could be classified as small at best ("d" = 0.01-0.31). These differences were slightly more pronounced for girls: ("d" = 0.01-0.34) and 13-18 year olds ("d" = 0.01-0.35). A statistical significant interaction between survey methods and gender was observed for Peer problems.

There was a tendency towards a smaller dispersion of self-report test scores in the phone survey compared to the mail survey. For the self-report scales Emotional and Peer problems, the SD-difference reached statistical significance. For the parent-reported scales the tendency was towards a slightly larger dispersion (SDs) of test-scores in the phone survey compared to the mail survey. Statistically significant differences were observed for the proxy report scales Peer problems and Prosocial.

As shown in Table 5, there were no systematic differences between mail and phone survey modes for the internal consistency Cronbach alpha values. In the mail survey, the alpha for the Total Difficulties self(proxy) score was 0.81(0.82) and ranged from 0.36(0.57) to 0.68(0.68) for the self-(proxy-) report subscales. For the phone survey, a Cronbach alpha of 0.73(0.82) was observed for the self-(proxy-) report SDQ Total difficulties score. For the subscales, the alpha values ranged from 0.34(0.35) to 0.66(0.80).

**Table 5:** Cronbach alpha and correlation between SDQ test scores (child's-self-report and parent proxy report)

		*Children's self-report*					*Parent's proxy-report*				
		EMO	CON	HYP	PEE	PRO	EMO	CON	HYP	PEE	PRO
		C1	C2	C3	C4	C5	P1	P2	P3	P4	P5
	*alpha*	*0.66*	*0.34*	*0.66*	*0.36*	*0.64*	*0.72*	*0.53*	*0.80*	*0.57*	*0.59*

C1	*0.66*		0.32	0.28	0.27	0.04	**0.31**	0.08	-0.01	0.16	0.03
C2	*0.36*	0.25		0.42	0.19	-0.29	0.21	**0.31**	0.28	0.14	-0.15
C3	*0.68*	0.27	0.30		0.16	-0.20	0.14	0.19	**0.31**	0.04	0.00
C4	*0.46*	0.31	0.17	-0.03		-0.18	0.16	0.10	0.13	**0.42**	-0.04
C5	*0.55*	0.10	-0.26	-0.16	-0.14		-0.03	-0.10	-0.18	-0.10	**0.22**

P1	*0.65*	**0.40**	0.15	0.20	0.19	0.04		0.31	0.34	0.33	-0.10
P2	*0.57*	0.10	**0.35**	0.27	0.08	-0.14	0.31		0.52	0.28	-0.28
P3	*0.79*	0.04	0.22	**0.47**	0.00	-0.13	0.37	0.51		0.29	-0.22
P4	*0.66*	0.11	0.09	0.01	**0.44**	-0.10	0.38	0.32	0.23		-0.19
P5	*0.68*	0.02	-0.15	-0.08	-0.09	**0.33**	-0.07	-0.37	-0.26	-0.23	

Lower triangular matrix = mail; upper triangular matrix = phone administration

^a ^statistically significant deviation after Fisher-Z Transformation;

EMO = Emotional; CON = Conduct; HYP = Hyperactivity; PEE = Peer Problems; PRO = Prosocial;

Alpha for the self-report Total Difficulties Score was 0.71 (mail) and 0.73 (phone);

Alpha for the Parent-report Total Difficulties Score was 0.82 (mail) and 0.82 (phone);

Correlation between Self-report and proxy report Total Difficulties Score was r = 0.43 (mail) and 0.37 (phone)

The SDQ MTMM analysis resulted in lower convergent and discriminant validity for the phone survey methods. The average mono-trait-multi-method correlation (between corresponding self- and proxy report scores) was r = 0.40 (range = 0.33-0.47) for the mail survey and r = 0.32 (range = 0.22-0.42) for the phone survey. The actual differences in r ranged from 0.01 to 0.16. Expressed in Fisher-Z values, the differences ranged from 0.02 to 0.19. The average multi-trait-mono-method correlation (across the self-report/proxy report version) was r = 0.20/0.32 for the mail survey. For the phone survey this value was r = 0.24/0.30 (self-report/parent report) on average and in many instances was similar or even higher than the desirable mono-trait-multi-method correlations. The multi-trait-multi-method correlation between non-corresponding self-report and proxy-report scales was r = 0.14 for both the mail and the phone survey (Table 5). The correlation between the self-and proxy Total difficulties score was r = 0.43 for the mail and r = 0.37 for the phone survey.

The SDQ measurement results were classified into the categories normal, borderline, and abnormal and cross-tabulated with the survey method. Statistically significant differences in the probability of a borderline or abnormal classification for the phone survey were tested using multinomial logistic regression. Table 6 shows the self-report, resulting in a smaller proportion and chance (OR) of abnormal classification for the dimensions Emotional (OR = 0.51), Conduct (OR = 0.47) and Peer problems (OR = 0.40). For the latter and the Prosocial dimension, a lower percentage was classified as borderline as well (OR = 0.66 and 0.46). For the proxy report version, the phone survey resulted in a higher chance of an abnormal classification in the SDQ dimensions Emotional (OR = 1.55) and Conduct (OR = 1.37). However, for the Prosocial dimension, a smaller chance of borderline (OR = 0.43) or abnormal (OR = 0.36) classification was seen. For the Total difficulties score, the phone survey was associated with a smaller chance of borderline classification (OR = 0.63).

**Table 6:** Multinomial logistic regression of the survey method on categorical SDQ classification (normal, abnormal, borderline)

	Mail		Phone							
	border-line	abnormal	border-line	abnormal	Raw OR phone borderline			Raw OR phone abnormal		
	**%**	**%**	**%**	**%**	**OR**	**95% CI**		**OR**	**95% CI**	

*Children's self-report*^*b*^	*n = 528-530*		*n = 498*							
Emotional	4.5	**5.3**	2.6	**2.8**	0.55	0.28	1.09	0.51*	0.26	0.97
Conduct	6.4	**4.2**	6.2	**2.0**	0.94	0.57	1.56	0.47^(*)^	0.22	1.00
Hyperactivity	7.9	9.5	8.6	8.4	1.08	0.69	1.69	0.89	0.58	1.37
Peer Problems	**12.3**	**3.8**	**8.6**	**1.6**	0.66*	0.44	0.99	0.40*	0.17	0.91
Prosocial^c^	**9.1**	2.1	**4.4**	2.2	0.46*	0.28	0.78	1.01	0.44	2.36
Total	8.7	3.2	7.6	1.6	0.85	0.54	1.33	0.48	0.21	1.13
*Parent's proxy-report*^*b*^	*n = 888-900*		*n = 791*							
Emotional	7.3	**6.1**	6.6	**9.2**	0.92	0.63	1.35	1.55*	1.08	2.24
Conduct	12.2	**11.6**	14.9	**14.7**	1.32	0.99	1.75	1.37*	1.03	1.83
Hyperactivity	4.2	7.7	5.7	7.0	1.36	0.87	2.12	0.91	0.63	1.32
Peer Problems	9.5	10.9	7.3	9.1	0.74	0.52	1.05	0.80	0.58	1.10
Prosocial^c^	**7.4**	**3.9**	**3.4**	**1.5**	0.43**	0.27	0.68	0.36**	0.19	0.70
Total	**8.1**	4.9	**5.2**	7.3	0.63*	0.43	0.94	1.48	0.99	2.23

^a ^Each SDQ dimension (categorical classification) served as the outcome in a multinomial regression with the survey method being the only covariate

^b ^Higher values indicate more strengths

## Discussion

This study investigated the effects of different interviewing methods on the measurement results for two different child and adolescent health instruments that assess different psychosocial constructs, the KINDL-R Quality of Life Questionnaire and the Strengths and Difficulties Questionnaire. A random selection of German children aged 8 to 18 was interviewed, half by telephone and half by mail. In line with other studies [7,9], telephone interviews led to a distinctly higher willingness of respondents to take part in the survey. This largely compensated for the fact that the chosen households were harder to contact due to the incompleteness of the public telephone records. Despite different coverage and response rates for the telephone and mail surveys, no significant differences were observed in the socio-demographic and socio-economic features of the two sub-samples, and thus both survey methods were composed of comparable samples. In addition, no specific interaction was found between the properties examined and the measurement results of the KINDL and the SDQ. The differences in the responses to these instruments ascertained in this study can therefore be attributed to the different methods of administration and to their interactions with other properties. For example, it is unlikely that families whose children had lower Quality of life/Mental Health were less likely to provide their phone number.

In line with adult surveys on the health-related quality of life instruments SF-8 [21] and SF-36 [20,2], many KINDL scales and SDQ scales display significantly more positive scores when ascertained over the telephone. The magnitude of the deviation could be classified as a small effect (0.2 of a SD), a finding similar to studies on the adult population using the SF-36 Health instrument [20].

Previous studies observed a more positive pattern of responses to questions of mental health by telephone as compared to mail, which might be explained by the greater sense of embarrassment or shame associated with reporting mental problems [20]. However, in this study we found the largest effects in scales measuring aspects of social functioning and social relations for both psychosocial constructs. Social desirability, e.g., being socially accepted, might play an important role as well. Limited cognitive processes (e.g., limited recollection performance in relation to telephone administration) might also account for differences in answering behaviour. For example, Brewer [38] showed that veterans predominantly reported severe (physical) symptoms on the telephone. The authors assumed that restricted answering time leads to a limited recognition of relevant information. Thus, the recollection under time pressure seems to be biased in favour of severe symptoms. Many aspects, symptoms or situations can be relevant for a valid rating. In the case of our instruments, assessing positive as well as negative symptoms would mean that the more pronounced positive as well as negative symptoms are reported. Statistical examination on item level (results not reported) showed a higher percentage of positively worded items being affected by survey mode differences compared to the negatively worded items. It could be that biased recognition performance favouring salient (positive and negative) symptoms over the telephone contributes to an overall more positive reaction to positively worded items. For negatively worded items, the biased recognition performance in favour of salient (negative) symptoms could conflict with mechanisms pointing in the opposite direction, such as social desirability.

Due to the smaller numbers of cases, the comparison of girls and boys for children and adolescents are less statistically powerful. The separate analyses can demonstrate effects of the survey method at a lower level of probability, but this limits the ability to interpret these effects. Available statistically significant results confirm the general findings. There was a tendency for differences between phone and mail methods to be more pronounced in adolescents than in children. For the SDQ the magnitude of the methods effect was also more pronounced in girls. Based on the present results, gender does not play a systematic role in the magnitude of the effect of the survey method for the KINDL.

The examination of internal consistency, reliability of item responses and convergent and discriminant validity shows substantially lower reliability and validity for the phone survey method. The decrease in the MTMM-convergent validity and the weaker discriminant validity was similar for both the KINDL-R and the SDQ and is mainly attributable to a lower convergence between children's self-reports and corresponding proxy ratings. Further research is required to elucidate the mechanism for this decrease in correspondence. Interestingly, the decrease in internal consistency of item-responses (when administered via phone) applied mainly to the KINDL-R. Again, in-depth research on the level of item response is warranted. The three response categories of the SDQ might be more suitable for phone administration, while the higher number (five) of response categories of the KINDL may be more difficult in phone interviews. Both instruments contain dimensions with positive and negatively worded items as well as dimensions with unmixed format. The decrease in internal consistency seems to be less pronounced in dimensions without such a reversal. Still, further research on which sort of item response categories are suitable for phone administration is needed.

## Conclusions

In conclusion, the use of different methods of administration leads to some differences in the test scores gathered from the KINDL-R and the SDQ beyond those attributable to chance alone. That is, the assessment of both constructs is affected by the survey method used. In general, the effects are small in magnitude, but these effects appear to be different among children and their parents for some of the domains. Despite being relatively small, the effects could bias results when comparing children and parents, and could be of practical relevance when looking at changes over time or in the estimation of prevalence of mental health problems. Though this study is not able to thoroughly determine which method of administration leads to the more valid, more reliable and least biased measurements, the actual results hint at lower reliability and validity of measurements from the phone survey method. This issue should and will be addressed by future studies. Based on a similar study, Ware et al. [20] recommend the use of corrective measures for the SF-36 instrument when carrying out telephone interviews. However, for our study, further research is warranted on the nature of the differences and the factors associated with such differences. In addition to the results presented, an analysis of differential item functioning (DIF) will be carried out, and we will examine whether items perform differently for a given construct of the scale depending on whether they are administered by phone or post. The ongoing studies will give additional insight into the question of whether the phone/post effect is general or due to specific items, such as the way items are worded. Until more in-depth research on a item-response level is conducted, no recommendations for suitable corrections will be made.

Future research is also warranted to determine how to handle the lower reliability in phone surveys. Generally speaking, deviations between telephone and mail surveys must be anticipated when using the KINDL-R and the SDQ. A direct comparison of results is therefore only possible with certain restrictions. In general, the adaptation of existing paper and pencil questionnaires to phone administration has to be done carefully and demands thorough testing if the psychometric quality of the original instrument is to be retained. It is important to note the survey mode effects on, for example, the internal consistency of item responses, which could differ between different psychosocial constructs and measures.

## Competing interests

The authors declare that they have no competing interests.

## Authors' contributions

ME conducted the statistical analyses and conceptualised and wrote the manuscript. RMW organised the study, trained the telephone interviewers and drafted a preliminary text version. AK assisted in the organisation of the study and the statistical analyses. URS was the principal investigator of the study, led the study and advised the conceptualisation and writing of the manuscript, as well as the statistical analysis plan. All authors read and approved the final manuscript.

## Pre-publication history

The pre-publication history for this paper can be accessed here:

http://www.biomedcentral.com/1471-2458/9/491/prepub
